# Effects of gastrointestinal tissue structure on computed dipole vectors

**DOI:** 10.1186/1475-925X-6-39

**Published:** 2007-10-22

**Authors:** Travis M Austin, Liren Li, Andrew J Pullan, Leo K Cheng

**Affiliations:** 1 Bioengineering Institute, The University of Auckland, Private Bag 92019, Auckland 1142, New Zealand; 2 Department of Engineering Science, The University of Auckland, Private Bag 92019, Auckland 1142, New Zealand

## Abstract

**Background:**

Digestive diseases are difficult to assess without using invasive measurements. Non-invasive measurements of body surface electrical and magnetic activity resulting from underlying gastro-intestinal activity are not widely used, in large due to their difficulty in interpretation. Mathematical modelling of the underlying processes may help provide additional information. When modelling myoelectrical activity, it is common for the electrical field to be represented by equivalent dipole sources. The gastrointestinal system is comprised of alternating layers of smooth muscle (SM) cells and Interstitial Cells of Cajal (ICC). In addition the small intestine has regions of high curvature as the intestine bends back upon itself. To eventually use modelling diagnostically, we must improve our understanding of the effect that intestinal structure has on dipole vector behaviour.

**Methods:**

Normal intestine electrical behaviour was simulated on simple geometries using a monodomain formulation. The myoelectrical fields were then represented by their dipole vectors and an examination on the effect of structure was undertaken. The 3D intestine model was compared to a more computationally efficient 1D representation to determine the differences on the resultant dipole vectors. In addition, the conductivity values and the thickness of the different muscle layers were varied in the 3D model and the effects on the dipole vectors were investigated.

**Results:**

The dipole vector orientations were largely affected by the curvature and by a transmural gradient in the electrical wavefront caused by the different properties of the SM and ICC layers. This gradient caused the dipoles to be oriented at an angle to the principal direction of electrical propagation. This angle increased when the ratio of the longitudinal and circular muscle was increased or when the the conductivity along and across the layers was increased. The 1D model was able to represent the geometry of the small intestine and successfully captured the propagation of the slow wave down the length of the mesh, however, it was unable to represent transmural diffusion within each layer, meaning the equivalent dipole sources were missing a lateral component and a reduced magnitude when compared to the full 3D models.

**Conclusion:**

The structure of the intestinal wall affected the potential gradient through the wall and the orientation and magnitude of the dipole vector. We have seen that the models with a symmetrical wall structure and extreme anisotropic conductivities had similar characteristics in their dipole magnitudes and orientations to the 1D model. If efficient 1D models are used instead of 3D models, then both the differences in magnitude and orientation need to be accounted for.

## Background

The stomach and small intestine have a common wall structure that consists of alternating layers of smooth muscle (SM) and pacemaker interstitial cells of Cajal (ICCs) [1]. The wall of the small intestine, in particular, is commonly represented by an outer longitudinal muscle (LM) layer and an inner circular muscle (CM) layer. These layers are separated by the myenteric plexus that contains the ICCs. ICCs are also found within the CM layer but their exact role is still uncertain.

Two basic patterns of electrical activity are present in the small intestine: slow waves and action potentials [1]. Slow waves are spontaneous rhythmic oscillations of the transmembrane potential. They have been shown to initiate in the ICCs and then conduct to smooth muscle cells via gap junctions [2]. At the peak of a slow wave, action potentials (sometimes referred to as 'spiking activity') can be triggered to generate a contractile response. Slow wave shape, frequency, amplitude and duration vary in different species and in different parts of the GI tract. In the human small intestine, slow wave frequency is around 12 cycles per minute (cpm) in the duodenum and decreases gradually to around 8 cpm at the terminal ileum [3,4].

In this article we only consider slow wave activity in the small intestine with the aim of improving the understanding of motility diseases associated with electrical disorders such as gastroparesis and myoelectrical dysrhythmia [5,6]. Simultaneously there is ongoing research into using Super Quantum Interference Devices (SQUIDs) to non-invasively measure the magnetic field of the small intestine and then use that information to characterise the underlying electrical fields in the intestine [7,8]. To interpret such recordings, however, requires understanding of what a normal magnetic field is in contrast to an abnormal magnetic field. Modelling has the potential to bring significant insight into this problem.

Dipoles are commonly used to represent the net electrical activity within a section of tissue or organ [9-12]. However, it is less certain how to relate the different dipole configurations back to the underlying electrical waveforms. This is especially true in the small intestine due to the alternating muscle layers and high regions of curvature.

Previous studies have shown that the intestinal dipoles may point at an angle to the intestinal wall rather than down the length of the intestine in the gross direction of the electrical activity [12]. It was postulated that the potential gradient through the intestinal wall was responsible for this behaviour. To help understand this behaviour further, we have explored similar calculations to [12] on a simpler geometry to carefully separate observed solution behaviour into its dependency on potential gradients through the intestinal walls and its dependency on geometric curvature.

In this study, we explore the contributing effects of different parameters on the magnitude and orientations of resultant equivalent dipole sources. These simulations have been performed on simplified geometries (a one-dimensional structure and a three-dimensional cylinder both with a straight and bent sections) which are topologically similar to short sections of a small intestine. This allowed the effects of curvature on the dipole parameters to be isolated. The effects of 1D and 3D meshes and varying the relative thickness of muscle layers and conductivity values are presented.

## Methods

### Simple geometries

**Figure 1:**
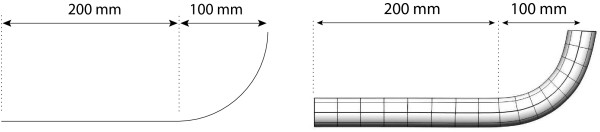
**Geometric meshes**. Simple geometric representations of an intestine composed of a straight and curved section. The 1D mesh consists of two sets of cell types occupying the same physical space and is represented by 16 cubic Hermite elements. The 3D model is a thin walled cylinder and is represented by a total of 1024 tricubic Hermite elements and contains three layers of different cell types (see Figure 2). The meshs are orientated such that the Y-axis runs vertically and Z-axis horizontally.

Simple 1D and 3D geometries (shown in Figure 1) representative of short sections of the small intestine were investigated. The gross geometry of these sections was described using cubic Hermite interpolation. Within each of these high-order coarse meshes a high resolution trilinear finite element mesh was constructed to solve the monodomain equations (see Equation 1) that govern the electrical propagation through the tissue [13]. In both cases the meshes are orientated such that the Y-axis is orientated vertically and Z-axis horizontally as shown in Figure 1.

The 1D model consisted of two 1D structures (occupying the same physical space) that represented the SM layer and the ICCs in the small intestine. Each structure was composed of a 200 mm straight section, followed by a 90 degree arc with a radius of approximately 100 mm. The geometry of each structure was represented by 16 cubic Hermite finite elements, with each element subsequently discretised using a high resolution mesh of linear finite elements, yielding a computational resolution of approximately 1 mm and a total of 602 solution points. This level of resolution was chosen since it was previously observed that numerical convergence was achieved when the mesh size approached 1 mm [12].

The 1D model was only capable of representing a slow wave propagating longitudinally down the intestine and as such could not capture any gradient of the slow wave through the intestinal wall. The 3D model, shown in Figure 1(right), was able to represent a transmural potential gradient as it contained a transmural thickness. The inner and outer radii of the 3D cylinder were 15 mm and 16.23 mm respectively, and the complete mesh was composed of a total of 1024 tri-cubic Hermite elements (16 elements in the longitudinal direction, 8 element circumferentially, and 8 element radially).

**Figure 2:**
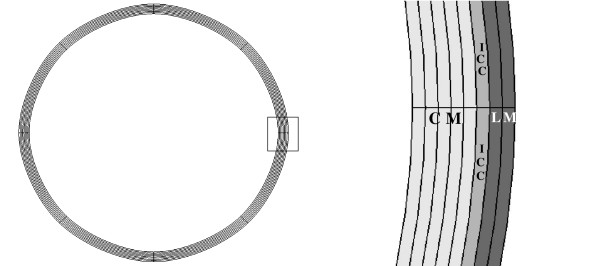
**Cross section of 3D mesh**. Cross section of the wall of the 3D cylindrical model (left) and an enlarged view of the eight element layers (right) through the intestinal wall. These eight layers in turn were grouped into three cell types: the LM, ICCs and CM, with an initial thickness ratio of 2:1:5.

The tissue structure of the small intestine was represented by assigning different properties to the different layers through the wall. As shown in Figure 2, the outermost two layers of elements represented the LM, the next layer the ICC, and the innermost five elements layers represented the CM. A similar ratio had previously been used in the anatomical model of the duodenum [12] and was an approximation to the real microstructure of the intestinal wall [2]. A high resolution hexahedral computational mesh was then defined in each geometric element resulting in a total of 174,624 computational points (with a resolution of ~3 mm, ~1 mm, and ~1 mm in each of the principal directions).

### The monodomain formulation using the Aliev cell model

Similar to what has been used in previous studies [12,14], the continuum-based monodomain model defined in Equation 1 was used to simulate the propagation of slow wave activity.

∇⋅(σ∇Vm)=Am(Cm∂Vm∂t+Iion)
 MathType@MTEF@5@5@+=feaafiart1ev1aaatCvAUfKttLearuWrP9MDH5MBPbIqV92AaeXatLxBI9gBaebbnrfifHhDYfgasaacH8akY=wiFfYdH8Gipec8Eeeu0xXdbba9frFj0=OqFfea0dXdd9vqai=hGuQ8kuc9pgc9s8qqaq=dirpe0xb9q8qiLsFr0=vr0=vr0dc8meaabaqaciaacaGaaeqabaqabeGadaaakeaacqGHhis0cqGHflY1cqGGOaakiiGacqWFdpWCcqGHhis0cqWGwbGvdaWgaaWcbaGaemyBa0gabeaakiabcMcaPiabg2da9iabdgeabnaaBaaaleaacqWGTbqBaeqaaOGaeiikaGIaem4qam0aaSbaaSqaaiabd2gaTbqabaGcdaWcaaqaaiabgkGi2kabdAfawnaaBaaaleaacqWGTbqBaeqaaaGcbaGaeyOaIyRaemiDaqhaaiabgUcaRiabdMeajnaaBaaaleaacqWGPbqAcqWGVbWBcqWGUbGBaeqaaOGaeiykaKcaaa@4DC5@

Here *σ *is the tissue conductivity tensor, *V*_*m *_is the transmembrane potential, *A*_*m *_is the surface to volume ratio of the continuum cell, *C*_*m *_is the membrane capacitance and *I*_*ion *_is the sum of the ionic currents from an appropriate cell model.

In all simulations the cellular ionic current term (*I*_*ion*_) was represented using the Aliev cell model [15]. The excitation parameter *ε *of the cell model was defined for the ICCs as linearly decreasing from 0.0825 at the proximal end to 0.067 at the distal end to mimic the frequency gradient of slow waves in the ICC layer of the duodenum in experimental observations [15]. Unless specified the parameters were the same as those presented in [12] and [15].

In previous work, isotropic conductivities were assumed for the LM, ICC, and CM layers [12]. The values used in the LM and CM layers were 0.4 mS mm^-1 ^and the value used in the ICC layer was 0.04 mS mm^-1^. Specific conductivity values across the various layers were defined according to [15]. Besides isotropic conductivity values, anisotropy conductivity values in the LM and CM layers were also investigated. Muscle fibres in both layers have limited electrical coupling between fibres and hence a lower degree of conduction. There is relatively little experimental data on intestinal anisotropy ratios, however, one such study has determined a longitudinal to circumferential propagation velocity ratio of 0.8 in a feline duodenum [16]. To examine the effects of such preferential pathways we reduced the degree of conductivity along and across the layer by one and two orders of magnitude to help understand the dipole vector dependency on the degree of anisotropy.

### Dipole vector computation

To simulate the electrical field of the torso and the magnetic field external to the torso, dipole vectors were used as source terms (*J*_*s*_) to represent intestinal electrical activity. This was computed using

*J*_*s *_= -*σ*(∇*V*_*m*_)

where *σ *is the conductivity tensor and *V*_*m *_is the transmembrane potential described previously.

The main part of computing J_*s *_is the calculation of the gradient vector, ∇*V*_*m*_. This vector was computed through the use of the local coordinate system and the known local-to-global coordinate mapping. We computed ∂*V*_*m*_/∂*ξ*_*i *_in the local space, for *i *= 1, 2, 3, and then inverted the local-to-global map to get an appropriate gradient, *i.e*.,

(∂Vm/∂x∂Vm/∂y∂Vm/∂z)=G−1(x,ξ)(∂Vm/∂ξ1∂Vm/∂ξ2∂Vm/∂ξ3)
 MathType@MTEF@5@5@+=feaafiart1ev1aaatCvAUfKttLearuWrP9MDH5MBPbIqV92AaeXatLxBI9gBaebbnrfifHhDYfgasaacH8akY=wiFfYdH8Gipec8Eeeu0xXdbba9frFj0=OqFfea0dXdd9vqai=hGuQ8kuc9pgc9s8qqaq=dirpe0xb9q8qiLsFr0=vr0=vr0dc8meaabaqaciaacaGaaeqabaqabeGadaaakeaadaqadaqaauaabeqadeaaaeaacqGHciITcqWGwbGvdaWgaaWcbaGaemyBa0gabeaakiabc+caViabgkGi2kabdIha4bqaaiabgkGi2kabdAfawnaaBaaaleaacqWGTbqBaeqaaOGaei4la8IaeyOaIyRaemyEaKhabaGaeyOaIyRaemOvay1aaSbaaSqaaiabd2gaTbqabaGccqGGVaWlcqGHciITcqWG6bGEaaaacaGLOaGaayzkaaGaeyypa0dcbeGae83raC0aaWbaaSqabeaacqGHsislcqaIXaqmaaGccqGGOaakcqWF4baEcqGGSaaliiWacqGF+oaEcqGGPaqkdaqadaqaauaabeqadeaaaeaacqGHciITcqWGwbGvdaWgaaWcbaGaemyBa0gabeaakiabc+caViabgkGi2IGaciab957a4naaBaaaleaacqaIXaqmaeqaaaGcbaGaeyOaIyRaemOvay1aaSbaaSqaaiabd2gaTbqabaGccqGGVaWlcqGHciITcqqF+oaEdaWgaaWcbaGaeGOmaidabeaaaOqaaiabgkGi2kabdAfawnaaBaaaleaacqWGTbqBaeqaaOGaei4la8IaeyOaIyRae0NVdG3aaSbaaSqaaiabiodaZaqabaaaaaGccaGLOaGaayzkaaaaaa@6E0D@

where **x **= (*x*, *y*, *z*)^*T*^, ***ξ ***= (*ξ*_1_, *ξ*_2_, *ξ*_3_)^*T*^, and **G**(**x**, *ξ*) is the Jacobian of the mapping from **x **to ***ξ***. Each ∂*V*_*m*_/∂*ξ*_*i *_is computed using simple first-order finite differences.

Along with the gradient we used a homogenised set of conductivity values to obtain J_*s*_. Normally *σ *is a full tensor and may even have discontinuities as is the case between the smooth muscle layers and the ICCs. At each solution point a dipole vector was computed that depended on the conductivities at that point and the solutions values at surrounding points. To acquire a lumped dipole source, dipole vectors at solution points were summed over a specified region, such as a geometric element or the entire domain, to create the desired number of lumped sources [14]. In the following studies, a single net dipole with a varying centre was always used (*i.e*., dipole contributions were vectorially summed over the entire domain). However, in most cases, the movement of the dipole centre was small compared with its magnitude.

## Results

The initial conditions of the transmembrane potential *V*_*m *_in the Aliev cell model are at its resting potential, *V*_*r *_(*i.e*., all cells are at rest). The simulation was run for a period long enough to allow the model to approach a steady-state frequency of 8 cpm that is typically observed in the terminal ileum of the human intestine. The simulation approached 8 cpm after 80 s of simulation time, at which point dipole vectors were then computed over a period of 8 s corresponding to the period of a single slow wave.

### Dipole vector in 1D model

**Figure 3:**
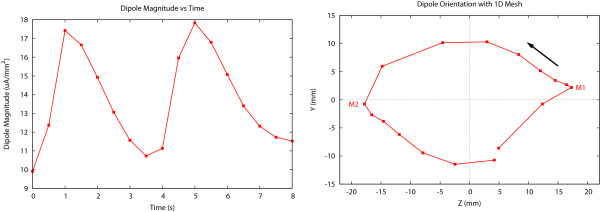
**1D mesh dipole magnitude and orientation**. Dipole magnitude (left) and orientation (right) computed using a 1D mesh over a period of 8 s. Over this time period a slow wave appears at the proximal and disappears at the distal end. The first and second maxima in the dipole magnitude, which occur at M1 and M2, are orientated along the principal axis of the intestine, with the solid black arrow indicating the direction of movement of the dipole head. Note that the intial dipole orientation is in the negative Y direction due to a previous wave repolarising at the distal end of the mesh.

The magnitude of the overall dipole vector in 1D changed cyclically with a period of approximately 8 s, corresponding to the duration of a single slow wave (depolarisation and repolarisation). Figure 3(left) displays the magnitude of the dipole vector over one period. It shows that there are two peaks in the magnitude, and the maximum is approximately 17 *μ*A mm^-2 ^and the first peak corresponded to the activation wave front and the second peak the repolarisation phase.

The dipole orientations over the same period are plotted in Figure 3(right). The curve traced the end of the dipole vector and the dipole centre was treated as fixed at the origin. It can be seen that the dipole rotated anti-clockwise during one period, due to the curvature and the propagation of the wave front. At the first maximum, the dipole appeared almost horizontal, pointing from the proximal end to the distal end of the mesh. This corresponded to a slow wave just appearing from the proximal end. When the wave was in the middle of the mesh, there were opposing dipole contributions from both depolarisation and repolarisation activity with the deloparisation wave front dominating. At the second maximum magnitude, the dipole reversed direction, corresponding to the same slow wave disappearing from the distal end.

### Effect of thickness ratio

The 1D model simulated the diffusion of current down the length of the intestine and between the ICC and SM layers. In the 3D models, the smooth muscle layer was decomposed into a CM and LM layer, and the effect of current diffusion through the wall and the significance of intestinal wall structure were investigated. In this section we examine the effect of the thickness of the CM muscle layer, under the assumption of isotropic conductivity for both the LM and CM layers. In each simulation the reference time of 0 s was chosen for each simulation such that it corresponded to the start of the slow wave.

**Figure 4:**
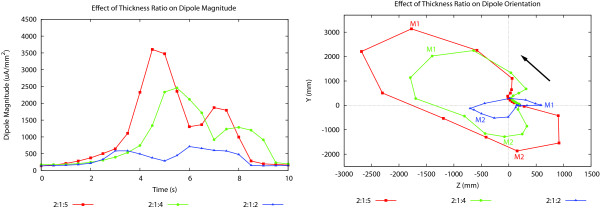
**Effect of thickness on dipole magnitude and orientation**. Effect of thickness of the CM layer on dipole (left) magnitude and (right) orientation computed using a 3D mesh over a period of ~10 s. Over this time period a slow wave appears from the proximal end of the mesh, propagates down the cylinder and disappears from the distal end. The maxima for each problem is denoted by M1 and M2 in (right), with the solid black arrow indicating the direction of the movement of the dipole heads. The ratios show the relative thicknesses of the LM, ICC and CM layers.

As shown in Figure 2, the original thickness ratio between the LM, ICCs and CM layers was set to be 2:1:5 (*i.e*., the outermost two element layers were assigned to be the LM, a single element layer underneath the LM was assigned to be the ICCs, and the remaining five element layers were assigned to be the CM). To examine the effect of varying the thickness of the CM layer, the thickness was reduced to four and two layers thick (resulting in ratios of 2:1:2 and 2:1:4). The dipole magnitude and orientations for these simulations are presented in Figure 4.

Figure 4(left) compares the magnitudes of the dipole vectors from these three simulations over a period of a slow wave. In each simulation there were two peaks, a larger peak corresponding to the front of the depolarisation wave and a smaller corresponding to the rear of the repolarisation activity. Similar behaviour was seen between the three models, with the large muscle volumes resulting in higher dipole magnitudes.

There were always two peaks in the magnitudes of both dipole vectors. For the model with the largest asymmetrical wall structure (thickness ratio 2:1:5), the first maximum was around 3500 *μ*A mm^-2^, while the second maximum was between 1500 and 2000 *μ*A mm^-2^. For the symmetrical model (thickness ratio 2:1:2), both maxima were between 500 and 1000 *μ*A mm^-2^, and the first maximum was slightly smaller than the second one. The decrease in magnitude was partly due to a decrease in the thickness of the wall, since dipole computation was weighted by volume.

The orientations of the dipoles are shown in Figure 4(right). They are significantly different to the relatively symmetric 1D results shown in Figure 3 (right). The symmetric model with muscle ratios of 2:1:2 is most similar to the 1D simulations. As the thickness of the CM layer increased, the dipole path became increasingly oblique, with an increased y-component.

**Figure 5:**
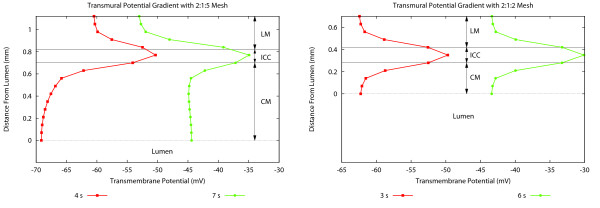
**Transmural potential gradients**. Transmural transmembrane potential for different muscle layer ratios at two time instances corresponding to the two dipole magnitude maxima shown in Figure 4. The model with muscle layer ratios of 2:1:5 shows an asymmetric profile across the wall, while the 2:1:2 models is symmetric. The asymmetric profile causes the dipole vector to be orientated at an angle relative to the principal direction of the slow wave. A 2:1:4 ratio yields a similar asymmetric behaviour to the 2:1:5 ratio but less pronounced.

In Figure 5, the transmural potential at the bend was plotted for two time periods which corresponded to the two maximum dipole magnitudes shown in Figure 4. These two times corresponded to instances when only the depolarisation or repolarisation fronts were present in the specimen. It was evident that unequal thicknesses of the smooth muscle layers led to asymmetrical transmural potential gradients on both sides of the ICCs, and hence a net transmural potential gradient across the wall. Due to unequal element volumes at the bend, the transmural potential gradient at the bend significantly contributed to the orientations of the dipoles. For the asymmetrical model, when the dipole was at a maximum magnitude, there was also a significant transmural gradient at the bend, resulting in an orthogonal dipole component.

It should be noted however, that the profile of the transmural electrical activity is contrary to that recorded experimentally in a canine antrum [17]. In this study it was shown that antral slow waves decrease in amplitude as they propagate through the circular muscle from the cells near the myenteric plexus to those near the submucosa.

### Effect of anisotropy in 3D bent cylinder

In the previous simulations, the smooth muscle layers were assumed to have isotropic conductivities. Although it is thought that in the LM and CM layers, the conductivity is likely to be highest in the longitudinal and circumferential directions (fibre directions) respectively, the effect of anisotropic conductivity tensor on dipole vector computation was not known. In the following section, this factor was investigated for the original model with thickness ratio of 2:1:5.

**Figure 6:**
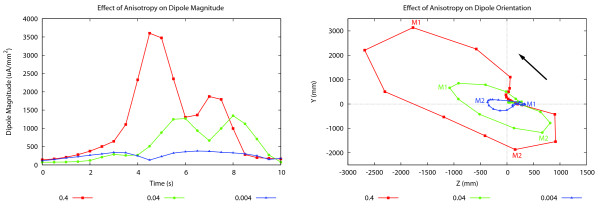
**Effect of conductivity on Dipole Magnitude and Orientation**. Effect of anisotropy on dipole vector (left) magnitude and (right) direction. The conductivity in the fibre direction was maintained at 0.4 mS mm^-1^, while the conductivity in the sheet and sheet-normal directions was varied between 0.4 mS mm^-1 ^and 0.004 mS mm^-1^. The maxima for each problem is denoted by M1 and M2 in (right), with the solid black arrow indicating the direction of the movement of the dipole heads.

For both smooth muscle layers, the conductivity in the principle fibre direction was kept at the original value of 0.4 mS mm^-1^, while the conductivities in the orthogonal directions (along and across the layers) were both reduced first to 0.04 mS mm^-1^, and then 0.004 mS mm^-1^. The magnitude and orientation of the dipole vectors for the different conductivities are shown in Figure 6.

The magnitude of the dipole vector decreased as the conductivities tranverse to the fibres were decreased, especially the two peaks. When the transverse conductivities were set to be 0.04 mS mm^-1^, the dipole magnitude at the two peaks were around 1200 *μ*A mm^-2^; when the same conductivities were reduced by another magnitude, the two peaks decreased to around 400 *μ*A mm^-2^. Generally, the first peak in the dipole magnitude still corresponded to the slow wave front near the proximal end of the bent cylinder, while the second peak corresponded to the same slow wave disappearing from the distal end. However, the decrease in conductivity across the intestinal wall in both smooth muscle layers led to a significant change in the transmural potential gradient at dipole peaks.

The orientation of the dipole also changed significantly, and the orthogonal component became less dominant as the tissue became increasingly anisotropic. Figure 6(right) shows that the decrease in dipole magnitude was much more significant in the y-direction, and orientations of the dipole at the peaks changed from being orthogonal in the original isotropic case to almost horizontal in the highly anisotropic case.

## Discussion

We have examined the effects of varying dimensionality, wall thickness ratio between the LM and CM muscle layers and conductivity on the resultant dipole magnitude and orientations using simplified geometries.

For the original 3D model (with a muscle thickness ratio of 2:1:5) the dipole magnitude was generally larger compared with the model with a symmetrical intestinal structure (thickness ratio 2:1:2). It was found that the greater the asymmetry between the LM and CM muscles the greater the dipole was orientated relative to the principal direction of the intestine. It was also found that both the bend and the transmural transmembrane potential gradient at the bend contributed to the components that resulted in oblique dipole angles. The transmural transmembrane potential gradient was caused by asymmetrical current diffusion through the LM and CM layers, and this increased as the asymmetry between the LM and CM layers increased. Since the thickness of the CM layer was greater than the LM layer, and dipole computation was weighted by volume, the potential gradients in the CM layer had a greater contribution to the magnitude and orientation of the dipole.

The peaks in the dipole magnitude in the original model resulted from the combinational effects of both longitudinal and transmural transmembrane potential gradients. The longitudinal gradient was due to slow wave propagation, while the transmural gradient was due to asymmetrical current diffusion through both smooth muscle layers. Generally, the simulated potential gradient was higher through the wall than along the intestine, so the peaks in dipole magnitude occurred when the transmural potential gradient at the bend was the highest, corresponding to a point in time when the front or back of the slow wave was passing through the bend.

Anatomical studies have shown fibre bundles in both the LM and CM layers with preferential conductivity along the fibre direction. The exact degree of anisotropy is likely to vary from subject to subject and between species. Two sets of conductivity values were used to investigate the effects of different degrees of anisotropy. As the conductivities transverse to the fibre direction were decreased, the overall dipole magnitude decreased also. This was partly because dipole computation is weighted by tissue conductivities according to Equation 2. In the CM layer, the potential gradient in the circumferential direction contributed the most to the overall dipole. Since the firing frequency of the ICCs depended only on the distance from the proximal end, around the circumference, all cells in the same depth should depolarise simultaneously. Consequently, the circumferential gradient was found to be minimal. As the conductivities in the other two directions were small, the contribution of the CM layer to dipole computation became much smaller. In the LM layer, where the fibre direction was along the cylinder, the longitudinal potential gradient was the most important. Therefore, the propagation of the slow wave in the LM layer played an important role in determining the magnitude and direction of the overall dipole.

In the original 3D model, orthogonal dipole components were a result of both the bend and the transmural potential gradient at the bend. With anisotropic conductivities, current diffusion through the wall was greatly affected since it was not aligned with the fibre direction. Therefore, as the muscle layers became increasingly anisotropic, the transmural gradient became less important for dipole computation. In the extreme case, dipole computation was mostly dependent on the longitudinal potential gradient in the LM layer.

The full 3D models are computationally expensive, requiring approximately 5 hours to solve on a single processor of a IBM Power 595 Power 5 computer. The 1D model however requires less than a minute to solve on the same computer. This difference in speed is largely due to the large increase in the number of computational points (approximately 174,000 for the 3D model compared with 600 for the 1D model) required to explicitly model the three layers and the wall thickness. Considering that the 3D model was only a short portion of a 6 m full intestine, solving for the slow wave activity in the entire intestine places a tremendous strain on most modern computing systems. One possibility may be to treat the intestine as a 1D curved structure and use rules to appropriately 'correct' the orientation of the dipole due to the lack of transmural potential gradient. However, it should be noted that there is a significant difference in dipole magnitude between the 1D and 3D models as shown in Figure 3 and the 2:1:5 simulation in Figure 4. This is due to the significantly reduced muscle mass (or number of cells) in the 1D model. If 1D models are used instead of 3D models, the differences in magnitude (as well as orientation) need to be accounted for.

It should be noted that our model has assumed that there are three discrete muscles layers, alternating between CM, ICC and LM cells. Although knowledge of structure of the musculature is still developing, our discrete layered representation is a simplification of the known structure. The ICCs have been found to be an intermingled network between the fibres of the CM and LM muscle layers, rather than discrete layers. The muscle layers are also believed to have different physiological roles in different locations in the GI tract and are likely to be different structurally [18]. We have also assumed that the Aliev cell model is an accurate representation of the cellular dynamics in the ICC and smooth muscle layers and in each our layers the cell types are assumed to be identical. These factors not included in our model may explain the reason our transmural electrical profile differs from that measured experimentally [17].

## Conclusion

We have systematically investigated a number of modelling parameters which effect the dipole magnitude and orientation when modelling a section of tissue with two smooth muscle and one ICC muscle layers. In this particular study we have used simplified geometries representative of a short section of the gastrointestinal tract, however, the findings are not restricted to this field.

It was found that dipole magnitude and orientation was effected by a variety of parameters. The structure of the intestinal wall affected the potential gradient through the wall and the resulting orientation and magnitude of the dipole vector. Although the 1D model was unable to represent transmural diffusion within each layer, it was able to represent the shape of the small intestine and successfully captured the propagation of the slow wave down the length of the mesh. We have seen that the models with a symmetrical wall structure and extreme anisotropic conductivities had similar characteristics in their dipole magnitudes and orientations to the 1D model.

As large 3D models of the intestine are computationally expensive, it may be possible in the future to use a more computationally efficient 1D model and adjust the dipole orientation and magnitude to account for dipole components missing as a result of the lack of transmural potential gradient.

## Competing interests

The author(s) declare that they have no competing interests.

## Authors' contributions

Authors TMA and LKC conceived the study, created and analyzed the final results. Author LL performed initial simulations and wrote an initial draft of the manuscript. Authors TMA, AJP and LKC were involved in the development of the study and editing of the final manuscript. All authors' read and approved the final version of the manuscript.
